# Arabidopsis PIZZA Has the Capacity to Acylate Brassinosteroids

**DOI:** 10.1371/journal.pone.0046805

**Published:** 2012-10-05

**Authors:** Katja Schneider, Christian Breuer, Ayako Kawamura, Yusuke Jikumaru, Atsushi Hanada, Shozo Fujioka, Takanari Ichikawa, Youichi Kondou, Minami Matsui, Yuji Kamiya, Shinjiro Yamaguchi, Keiko Sugimoto

**Affiliations:** 1 RIKEN Plant Science Center, Tsurumi, Yokohama, Kanagawa, Japan; 2 RIKEN Advanced Science Institute, Wako, Saitama, Japan; Instituto de Biología Molecular y Celular de Plantas, Spain

## Abstract

Brassinosteroids (BRs) affect a wide range of developmental processes in plants and compromised production or signalling of BRs causes severe growth defects. To identify new regulators of plant organ growth, we searched the Arabidopsis FOX (Full-length cDNA Over-eXpressor gene) collection for mutants with altered organ size and isolated two overexpression lines that display typical BR deficient dwarf phenotypes. The phenotype of these lines, caused by an overexpression of a putative acyltransferase gene *PIZZA (PIZ),* was partly rescued by supplying exogenous brassinolide (BL) and castasterone (CS), indicating that endogenous BR levels are rate-limiting for the growth of *PIZ* overexpression lines. Our transcript analysis further showed that *PIZ* overexpression leads to an elevated expression of genes involved in BR biosynthesis and a reduced expression of BR inactivating hydroxylases, a transcriptional response typical to low BR levels. Taking the advantage of relatively high endogenous BR accumulation in a mild *bri1-301* background, we found that overexpression of *PIZ* results in moderately reduced levels of BL and CS and a strong reduction of typhasterol (TY) and 6-deoxocastasterone (6-deoxoCS), suggesting a role of PIZ in BR metabolism. We tested a set of potential substrates *in vitro* for heterologously expressed PIZ and confirmed its acyltransferase activity with BL, CS and TY. The *PIZ* gene is expressed in various tissues but as reported for other genes involved in BR metabolism, the loss-of-function mutants did not display obvious growth phenotypes under standard growth conditions. Together, our data suggest that PIZ can modify BRs by acylation and that these properties might help modulating endogenous BR levels in Arabidopsis.

## Introduction

How characteristic size of organs is controlled in multicellular organisms is a fascinating and important question in biology. The final size of plant organs is determined by the balance between cell proliferation and cell differentiation. During organ growth, cells first proliferate through the mitotic cell cycle and increase biomass by supplying new cells into an organ. In the subsequent post-mitotic phase, cells differentiate and expand their volume through water uptake into the vacuole and cell wall biogenesis. This step is often associated with an alternative cell cycle called endoreduplication cycle or endocycle in which cells amplify the nuclear DNA content to increase their ploidy level [1]. Controlling the duration of cell proliferation and cell expansion or the transition from the proliferative phase to the expansion phase is crucial for the determination of final organ size (reviewed in [2]). Accumulating evidence suggest that these processes are controlled both transcriptionally and post-translationally, and several transcriptional regulators or enzymes involved in the post-translational protein modification have been shown to act on cell proliferation or cell expansion [3], [4].

The extent of cell proliferation and cell expansion is influenced by both developmental and environmental cues [5], and various plant hormones act as a transducer of these upstream signalling. Diverse ranges of mutants impaired in the biosynthesis or signalling of these plant hormones have been identified and as expected, many of them show defects in cell proliferation or cell expansion. Plants missing one of the BR biosynthesis genes, such as *CONSTITUTIVE PHOTOMORPHOGENENSIS* (*CPD*), *DWARF4* (*DWF4*) or *ROTUNDIFOLIA3* (*ROT3*), or BR perception genes, such as *BRASSINOSTEROID INSENSITIVE1* (*BRI1*), exhibit characteristic dwarf phenotypes with dark leaves and prolonged life span [6], [7]. Several enzymes of BR metabolism have also been identified, including sulfotransferases ST1 and ST4a (BNST3/4 in *Brassica napus*), a UDP glucosyltransferase UGT73C5 and P450 hydroxylases PHYB-4 ACTIVATION TAGGED SUPRESSOR 1 (BAS1) and SUPRESSOR OF PHYB-4 7 (SOB7). Recently, expression of a BAHD acyltransferase-like protein BRASSINOSTEROID INACTIVATOR 1 (BIA1) was shown to associate with changes in BR levels [8]. Plants overexpressing these genes display dwarf phenotypes that resemble mutants in BR biosynthesis and signalling [6], [9], [10], [11], suggesting that inactivation of BRs is also an important mechanism to control endogenous BR levels. In contrast to biosynthesis genes, however, the loss of BR metabolism genes does not appear to be associated with strong phenotypic changes.

Unlike animals, plants are often viable after strong alterations in organ size but highly redundant pathways controlling organ size have restricted the investigation of underlying molecular mechanism using loss-of-function mutants. To overcome these problems, we screened the FOX collection of Arabidopsis [12], [13] to identify novel regulators of organ growth. The strength of the FOX lines compared to previous activation tagging lines (e.g. [14]), is that they express normalized full-length cDNA under the control of the CaMV *35S* promoter, thus identifying causal genes by PCR is usually very straight-forward. The FOX system so far have led to the identification of novel transcription factors involved in stress tolerance [15], trichome cell expansion [16], nitrogen sensing and metabolism in Arabidopsis [17], [18] and the same over-expression system is now extended to analyse gene functions in rice [19], [20]. In this study, we isolated two dwarf mutant lines strongly resembling BR deficient plants. We found that these phenotypes are caused by an overexpression of the *PIZ* gene encoding a putative acyltransferase and that exogenous application of BL and CS partially rescues the dwarf phenotype. From our *in vitro* enzymatic assay, we show that PIZ proteins have an acyl-CoA ligase activity, producing a novel form of acylated BRs. Our data suggest that PIZ functions as a new enzyme in BR metabolism.

## Results

### Isolation of PIZ Overexpression Lines with a Dwarf Phenotype

**Figure 1:**
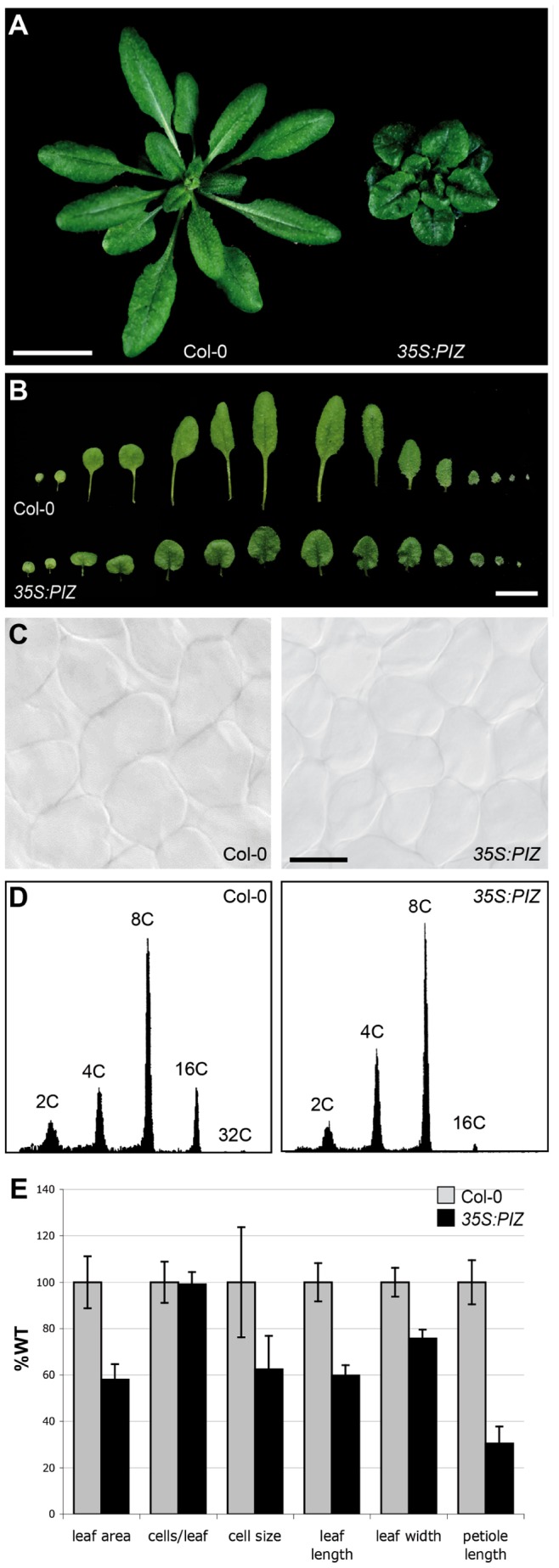
Overexpression of *PIZ* causes severe dwarf phenotypes. (**A**) 32-day-old wild-type (Col-0) and *35S:PIZ* seedlings. (**B**) Leaf size of 32-day-old Col-0 and *35S:PIZ* seedlings. (**C**) Light microscopic images of leaf mesophyll cells. (**D**) Flow cytometry analysis of first and second leaves from 17-day-old Col-0 and *35S:PIZ* seedlings. (**E**) Leaf and cell size of 21-day-old *35S:PIZ* seedlings. Values are shown relative to Col-0 and error bars represent ±SD of the means. n = 20 for leaves, n = 300 for cell size and n = 4 for cell number (p<0.0001, student’s t-test). Scale bars represent 1 cm in (**A**, **B**) and 50 µm in (**C**).

**Figure 2:**
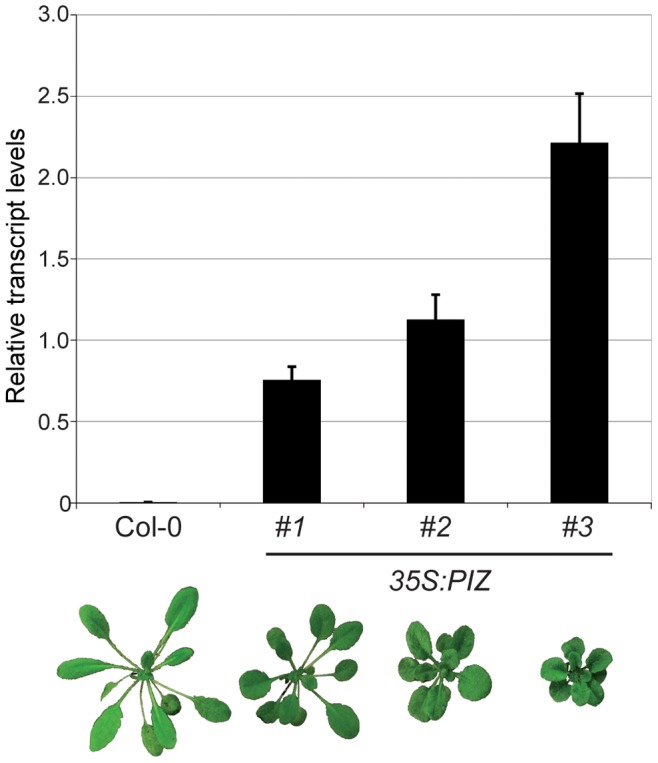
The *PIZ* transcript levels correlate with the severity of dwarf phenotypes in *35S:PIZ* plants. Quantitative PCR analysis of the *PIZ* transcripts in 21-day-old wild-type (Col-0) and weak, intermediate and strong *35S:PIZ* plants (from left to right). Mean expression values from two biological replicates are normalized against those of *PP2AA3* and shown with SD.

From a screen of the Arabidopsis FOX collection [12] for organ size mutants, we isolated two overexpression lines F23131 and F28215 that display typical BR deficient dwarf phenotypes with small round leaves of dark green colour (Figures 1A and 1B). These phenotypes segregated dominantly in the T_2_ generation and correlated with the presence of the transgene, suggesting that it is caused by insertion of a single gene. We recovered the cDNA inserted in both lines by PCR using primers specific for the FOX vector and found by sequencing analysis that both cDNAs encode the same gene (*At4g31910*) which we named *PIZZA* (*PIZ*) for the round leaf phenotypes of its overexpressor. The transgenic lines expressing *PIZ* cDNA driven by the CaMV *35S* (*35S*) promoter in wild-type background reproduced the dwarf phenotype identical to F23131 and F28215 plants (Figure 2), confirming that the phenotype of the FOX lines is caused by overexpression of the *PIZ* gene. Quantitative PCR analysis revealed that the severity of the phenotype correlates with the level of *PIZ* transcripts (Figure 2). We noticed silencing effects in later generations of the F28215 lines as well as of the retransformed lines, leading to intermediate to wild-type size of plants (data not shown). Since F23131 lines produced most stable phenotypes, this line, described as *35S:PIZ* hereafter, was used for further experiments.

**Figure 3:**
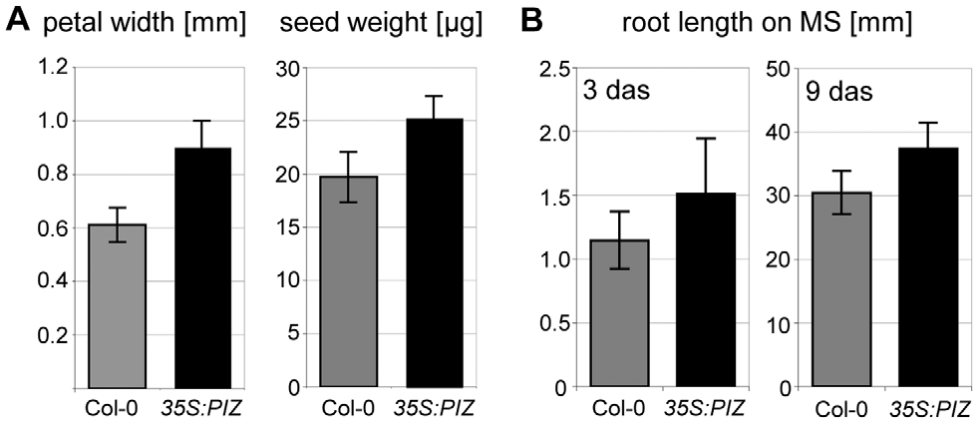
Growth of flowers, seeds and roots is enhanced in *35S:PIZ* plants. (**A**) Petal width and seed weight. Error bars represent ±SD of the means. n ≥ 35 for petal width (p<0.001, student’s t-test) and n = 3 (p<0.05, student’s t-test) for seed weight. (**B**) Root length of 3-day-old (left) and 9-day-old (right) seedlings, n≥22 (p<0.001, student’s t-test).

Our quantitative analysis showed that leaf area of 21-day-old *35S:PIZ* seedlings is reduced to about 60% of wild-type and this is caused by a decrease in cell size but not in number of cells per blade (Figures 1C and 1E). Similar to *rot3,* a previously reported BR biosynthesis mutant [21], the reduction of leaf length was more pronounced than that of leaf width and the strongest growth retardation was found on the petiole (Figure 1E). The ploidy level of first true leaves from 21-day-old *35S:PIZ* seedlings is slightly reduced compared to wild-type mainly due to a reduction of the 16C peak from 13.5±4.8% to 4.0±2.0% of total nuclear counts (n = 3) (Figure 1C). In contrast to leaves, petals of *35S:PIZ* flowers are wider than those of wild-type by about 148% without significant alterations in their length (Figure 3A). We found that *35S:PIZ* seeds are irregularly shaped and enhanced in average weight by 128% compared to wild-type (Figure 3A). Under continuous light conditions, *35S:PIZ* roots are about 132% and 123% longer compared to wild-type at 3 and 9 days after sowing, respectively (Figure 3B).

### Exogenous Application of BL and CS Partly Complements the Dwarf Phenotype of *35S:PIZ* Seedlings

**Figure 4:**
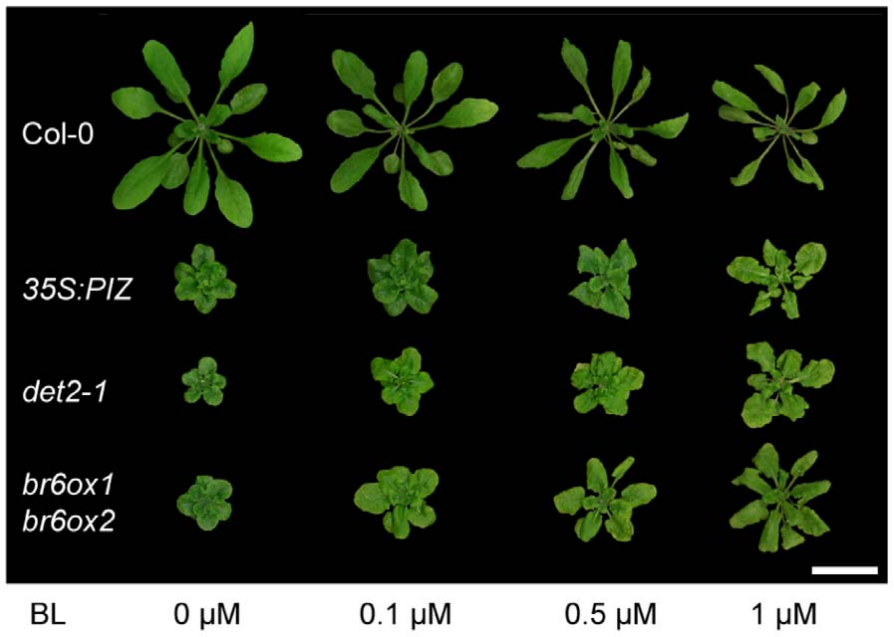
Exogenous BL partly complements the dwarf phenotype of 35S:*PIZ* seedlings. Plants grown on soil were sprayed every 4 days with an aqueous solution of BL at 0, 0.1, 0.5 and 1 µM for 4 weeks. Similar to BL biosynthesis mutants *det2-1* and *br6ox1 br6ox2*, the dwarf phenotype of *35S:PIZ* seedlings is partially rescued by exogenously applied BL.

While the *35S:PIZ* phenotype strongly resembles plants impaired in BR biosynthesis or signalling [21], [22], [23], [24], [25], [26] and those with enhanced inactivation of brassinolide or castasterone [7], [25], [27], defects in the production or response of gibberellic acid (GA) may also result in similar dwarf plants. Therefore, we first tested whether exogenously applied BR or GA complements the dwarf phenotypes of *35S:PIZ*. As shown in Figure 4, we found that the *35S:PIZ* phenotypes partially diminish by spraying an aqueous solution of 0.1 to 1 µM BL on the aerial tissues of plants grown on soil. Similarly, adding 0.1 to 1 µM of BL or CS to the growth medium recovered growth of *35S:PIZ* seedlings although compared to *deetiolated2-1* (*det2-1*) and *brassinosteroid-6-oxidase* (*br6ox1 br6ox2)* mutants impaired in BR biosynthesis [28], [29], 10 times higher dosages of CS or BL were needed for comparable effects (Figure S1). We also applied several other precursors of BL such as TY, TE and campesterol to the growth media, but none of these compounds, when applied at concentrations of up to 1 µM, rescued the *35S:PIZ* phenotype (data not shown). To test whether the *35S:PIZ* phenotype is caused by GA deficiencies, we added 0.1 µM GA_4_ to the growth media. While this concentration of GA_4_ was sufficient to complement the GA biosynthesis mutants *ga3ox1 ga3ox2* [30], it had no visible effects on the growth of *35S:PIZ* seedlings (Figure S1). These data support the idea that the endogenous BR levels are reduced in *35S:PIZ* plants and that this, at least in part, accounts for their dwarf phenotypes.

### Overexpression of PIZ Modifies the Expression of BR-related Genes

To further explore how an overexpression of *PIZ* causes the dwarf phenotype, we studied the genome-wide transcriptional changes in *35S:PIZ* plants using the Affymetrix ATH1 chip microarray. The transcript analysis of 21-day-old wild-type and *35S:PIZ,* where we see very strong reproducible growth retardation, confirmed that the endogenous expression of *PIZ* in wild-type in aerial tissues is marginal and the introduction of the *35S:PIZ* construct leads to a more than 200-fold increase in *PIZ* transcript levels (Table 1). After quality control for absolute expression values and consistency among the three biological replicates, additional 115 genes were identified with an expression level changed more than 1.5-fold in *35S:PIZ*. In general, these changes were relatively moderate and the expression of only 23 genes was changed more than 2-fold (Tables 1 and 2). These data are consistent with previous microarray experiments reporting that BR treatment has surprisingly restricted effects on gene expression compared to other phytohormones [31], [32].

**Table 1:** Genes up-regulated in *35S:PIZ* plants.

AGI code	gene	fold up	protein	process	localisation	LitBL
*At4g31910*	***PIZ***	231.8	acyl-transferase	BR metabolism	unknown	
*At5g01740*		7.17	Ketosteroid-isomeraserelated (s)	contains wound-induced WI12 domain (s)	unknown	0.1
*At5g57785*		4.37	unknown	unknown	unknown	0.0
*At4g36380*	***ROT3***	4.06	monooxigenase	leaf morphogenesis, BR synthesis	ER (e)	0.5
*At5g57780*		3.37	unknown	unknown	unknown	0.2
*At3g45600*	*TET3*	3.19	tetraspanin	aging (s)	integral to plasma membrane	
*At3g30180*	***BR6OX2***	3.10	monooxigenase	BR biosynthesis	endomembrane system (e)	0.1
*At4g01680*	*MYB55*	2.91	transcription factor (s)	regulation of transcription	nucleus (e)	0.2
*At3g28220*		2.32	unknown	induced by salt stress	vacuole, chloroplast envelope	
*At5g09440*	*EXL4*	2.29	Exordium like 4	cell expansion	membrane	0.7
*At2g26020*	*PDF1.2b*	2.00	defensin	xenobiotics metabolism, defence	extracellular, cell wall, endomembrane system (e)	

Transcript levels were compared between 3-week-old wild-type and *35S:PIZ* seedlings by microarray analysis. (s) inferred from sequence, (e) inferred from electronic annotation. Lit BL: Published transcriptional response to BL treatment [40].

**Table 2:** Genes down-regulated in *35S:PIZ* plants.

AGI code	gene	fold down	protein	process	localisation	Lit BL
*At2g28085*		5.15	unknown	auxin response (s)	unknown	
*At3g45590*	*ATSEN1*	4.35	subunit of tRNA-intron endonuclease (s)	t-RNA splicing	endonuclease complex	
*At3g45970*	*ATEXLA1*	3.94	expansin-like	anisotropic cell growth, cell wallloosening (e)	cell wall	3.9
*At2g26710*	***BAS1***	3.76	monooxygenase	BR metabolism, response to light stimulus	endomembrane system (e)	2.9
*At1g78970*	*LUP1*	3.09	lupeol synthase	pentacyclic triterpenoid biosynthesis	unknown	2.0
*At3g03830*	*SAUR28*	2.80	unknown	response to auxin stimulus (s)	unknown	2.1
*At5g41900*		2.78	hydrolase (s)	cuticle development (s), 75% amino acid identity to *bodyguard1 BDG1*	endomembrane system (e)	1.7
*At2g37130*	*PER21*	2.55	peroxidase/oxido-reductase (s)	defence, response to oxidative stress and fungus (s)	endomembrane system (e)	
*At5g12940*		2.35	protein binding (s)	defence (fungus), polysaccharidecatabolism (s)	endomembrane system (e)	
*At3g12500*	*ATHCHIB*	2.34	chitinase	defence (fungus), polysaccharide/chitincatabolism (s)	vacuole, plasma membrane	
*At4g16563*		2.31	aspartic-type endopeptidase (e)	proteolysis (s)	cell wall	1.5
*At5g02760*		2.24	serine/threonine phosphatase PP2C family (s)	protein amino acid dephosphorylation (s)	protein Ser/Thr phosphatase complex	
*At3g58120*	*BZIP61*	2.18	BZIP transcription factor	regulation of transcription, binds toAtbZIP34	nucleus	1.4

Transcript levels were compared between 3-week-old wild-type and *35S:PIZ* seedlings by microarray analysis. (s) inferred from sequence, (e) inferred from electronic annotation. Lit BL: Published transcriptional response to BL treatment [40].

*PIZ* overexpression causes a clear upregulation of several BR biosynthesis genes such as *ROT3* (4.06-fold) and *BR6ox2* (*CYP85A2* [29]) (3.10-fold), and a weaker induction of *CYP90D1* (1.91-fold), a close homolog of *ROT3* [7], [33] and *CPD* gene expression (1.62-fold) (Table 1). In contrast, we noted that the expression of a BR inactivation gene, a BL hydroxylase *BAS1,* is downregulated (−3.76-fold) in *35S:PIZ* (Table 2). The expression of other BR-regulated genes *DWF4*, *TOUCH4* (*TCH4*) and *BR ENHANCED EXPRESSION1* (*BEE1*) [34], [35] also changed with expected tendencies (1.44, −1.96 and 1.4-fold, respectively) but with relatively high variations among replicates (data not shown). As expected from the strong growth phenotype, several genes involved in cell expansion or cell wall modification were downregulated in *35S:PIZ* plants. These included expansin-like 1 [36] (*ATEXLA1*, −3.94-fold), cell wall loosening xyloglucan endotransglycosylase/hydrolase *XTH33* [37] (−1.84-fold) and receptor like kinase *THE1* [38] (−1.6) (Table 2). The latter two were recently shown to be induced by BL and downregulated in *bri1-5* mutants [39], [40]. Indeed, we found that more than 50% of the genes mis-regulated in *35S:PIZ* exhibit similar transcriptional responses to BL treatment (Tables 1 and 2). Interestingly, the majority of genes with altered expression encode proteins with enzymatic activities and we found very few genes implicated in signal perception or transduction. It should be also noted that other than BR-related genes, we found very few phytohormone-related genes and the only other genes that showed more than 2-fold difference in *35S:PIZ* were those implicated in auxin response (Table 2). We also noted that proteins localised to the endomembrane system are over-represented in both up- or downregulated categories (Tables 1 and 2).

**Figure 5:**
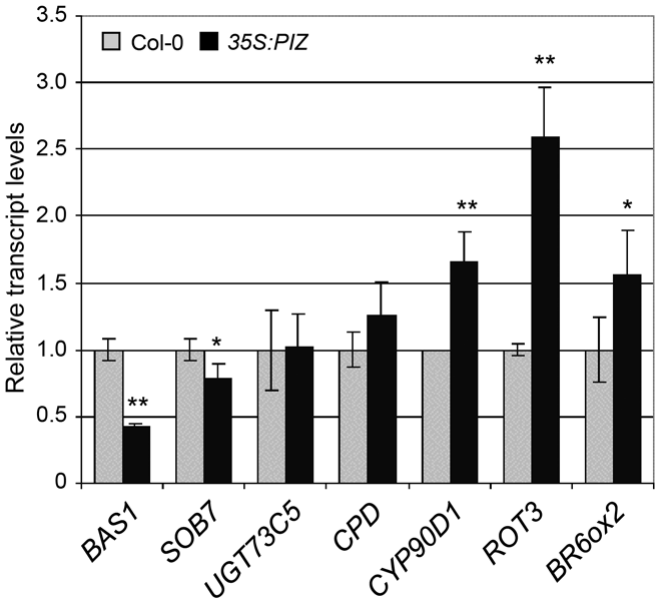
Overexpression of *PIZ* modifies the expression of BR related genes. Quantitative PCR analysis of *BAS1*, *SOB7* and *UGT73C5*, implicated in BR metabolism, and *CPD*, *CYP90D1*, *ROT3* and *BR6ox2*, involved in BR biosynthesis. RNA was isolated from aerial tissues of 17-day-old plants. Mean values of three biological replicates, normalised to *PP2AA3* and shown relative to Col-0 levels with SD. * and ** represent significant difference at p<0.1 and p<0.05, respectively.

By quantitative PCR we further tested the expression of selected genes with functions in BR metabolism and confirmed the down-regulation of *BAS1* (−2.3-fold) and *SOB7* (−1.3-fold) that act as a hydroxylase in BR inactivation (Figure 5). On the contrary, we did not find significant difference in the level of *UGT73C5,* a glycosyltransferase implicated in BR metabolism (Figure 5). We also confirmed the upregulation of four genes in BR biosynthesis with *ROT3* (2.6-fold), *CYP90D1* (1.65-fold) and *BR6ox2* (1.56-fold) showing the strongest induction. As reported for other BR deficient mutants [41], [42], these transcriptional changes in *35S:PIZ* may result from the feedback regulation in response to the reduced levels of endogenous BR.

### Metabolites from BR Biosynthesis are Reduced in *35S:PIZ* Seedlings

**Figure 6:**
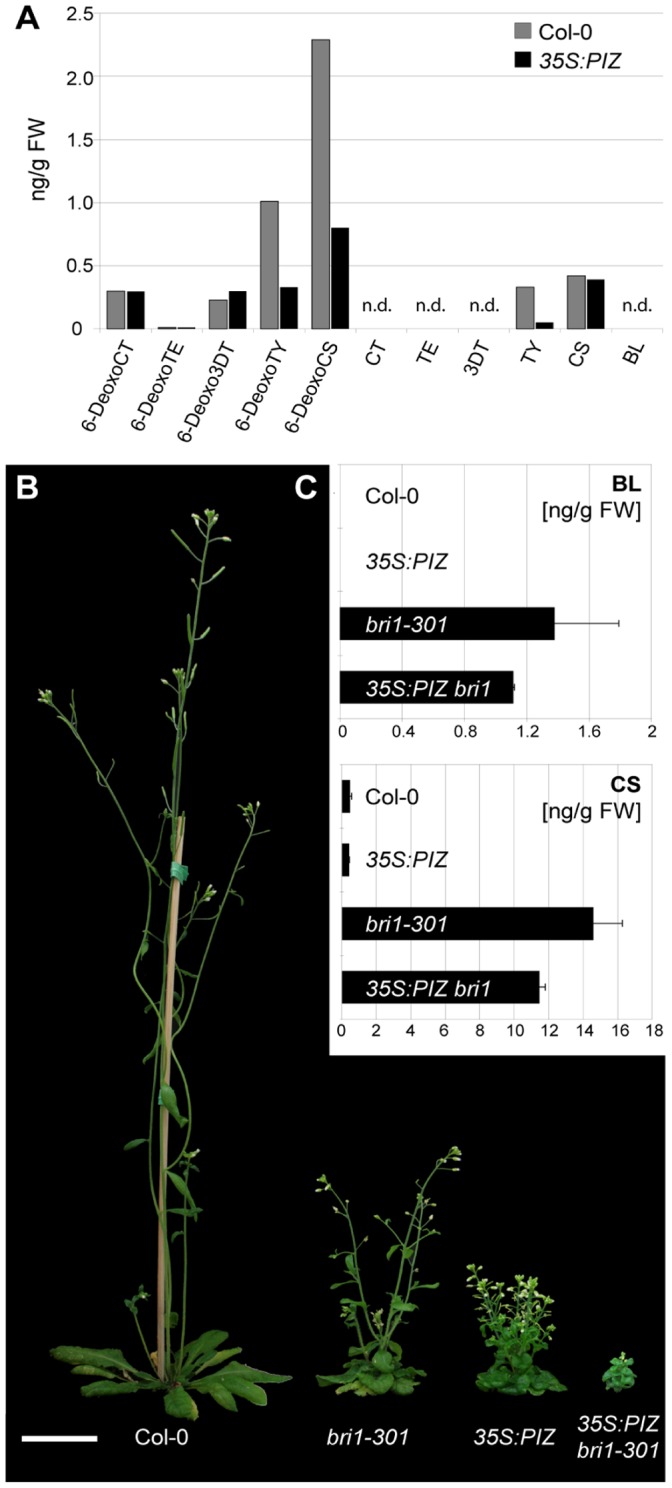
Endogenous BR levels in *35S:PIZ* and *35S:PIZ bri1-301* seedlings. (**A**) Free BR levels in aerial tissues of 4-week-old *35S:PIZ* seedlings quantified by GC-MS are shown in ng/g fresh weight (FW). 6-deoxo-cathasterone (6-deoxoCT), 6-deoxo-teasterone (6-deoxoTE), 3-dehydro-6-deoxoteasterone (6-deoxoDT), 6-deoxo-typhasterol (6-deoxo-TY), 6-deoxo-castasterone (6-deoxoCS), cathasterone (CT), teasterone (TE), 3-dehydro -teasterone (3DT), typhasterole (TY), castasterone (CS), brassinolide (BL). n.d., not detected. (**B**) Growth phenotypes of 17-day-old Col-0, *bri1-301, 35S:PIZ* and *bri1-301 35S:PIZ* (from left to right). Scale bar represents 3 cm. (**C**) Free BL and CS levels from aerial tissues of 17-day-old plants are analysed by LC-ESI-MS/MS. Mean values of three biological replicates are shown, relative to fresh plant weight (FW), with SD.

Our data suggest that ectopic overexpression of *PIZ* reduces endogenous BR levels, resulting in the dwarf phenotype similar to other BR deficient mutants. To directly test this hypothesis, we analysed a broad range of compounds from early steps of BR biosynthesis (compare e.g. [43]) by gas chromatography-mass spectroscopy (GC-MS). Compared to wild-type, endogenous levels of detectable BRs were generally low in 30-day-old *35S:PIZ* aerial tissues and the level of 6-deoxotyphasterol (6-deoxoTY), TY, 6-deoxoCS and CS was reduced to 33%, 15%, 35% and 93% of WT levels, respectively (Figure 6A, Figure S2). The level of most active BR, BL, was below the detection limit in both wild-type and *35S:PIZ.*

To further validate our hypothesis, we also quantified the CS and BL levels in aerial tissue of 17- to 21-day-old plants by liquid chromatography-electrospray-tandem mass spectrometry (LC-ESI-MS/MS). The level of BL was again too low for detection but consistent with the data from our GC-MS analysis, enhanced expression of *PIZ* led to a moderate but significant reduction of CS to 85.5±3.3% of wild-type level (Figure 6C). It was previously shown that the endogenous BR levels are highly elevated in *bri1-301*, a weak *bri1* mutant allele, in which impaired BR signalling provokes the feedback upregulation of BR biosynthesis [44]. Taking this as an advantage, we expressed the *35S:PIZ* construct in *bri1-301* and compared the BL and CS levels in *35S:PIZ bri1-301* background to those in *bri1-301* alone. As previously reported [45], the CS and BL levels were strongly increased in *bri1-301* and we detected 14.57±1.69 ng/gFW of CS and 1.37±0.43 ng/gFW of BL from plants grown under our growth condition (Figure 6C). The expression of *35S:PIZ* in the *bri1-301* background resulted in an enhancement of the growth phenotype (Figure 6B) and reduction of the endogenous CS and BL levels to 78.4±0.2% and 80.4±0.4%, respectively, of wild-type (Figure 6C).

### The PIZ Protein Sequence Possesses Features as an Acyltransferase

**Figure 7:**
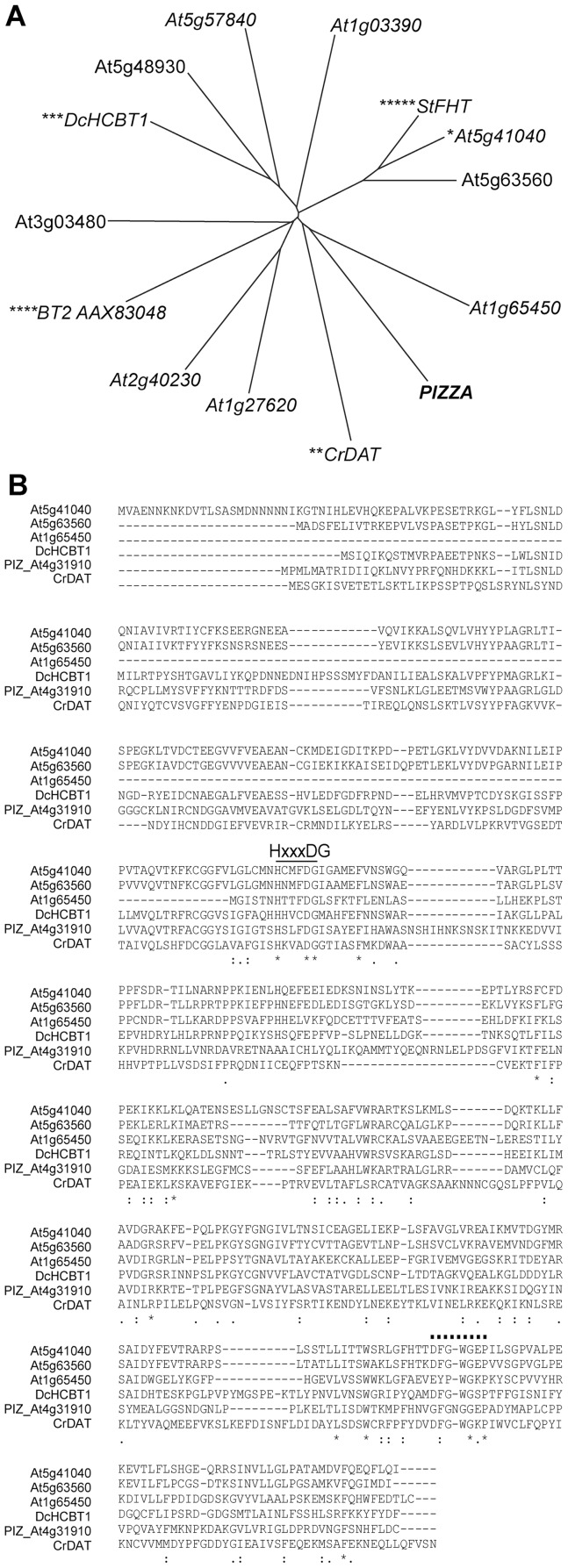
The *PIZ* gene structure and phylogenetic analysis. (**A**) The phylogenetic tree based on CLUSTALW alignment of protein sequences. *At5g41040, putative feruloyl-CoA transferase [*Arabidopsis thaliana*] with highest sequence similarity to PIZ. **CrDAT, deacetylvindoline 4-O-acetyltransferase [*Catharanthus roseus*]. ***DcHCBT1, anthranilate N-hydroxycinnamoyl/benzoyltransferase [*Dianthus caryophyllus*]. ****BT2 AAX83048, taxadienol acetyl transferase [*Ozonium* sp. BT2]. *****StFHT, BAHD feruloyl transferase [*Solanum tuberosum*]. (**B**) Amino acid sequence alignment of PIZ and its close relatives. Amino acid sequences are aligned using ClustalW. Underlined HxxxDG indicates an acyl transferase active site and dotted line marks the consensus DFGWGKP sequence conserved among these transferases.

Our amino acid sequence analysis using the InterProScan software grouped PIZ into the Interpro family IPR003480 as a CoA-dependent acyltransferase (EC:2.3.1, Pfam motif PF02458) transferring acyl groups other than amino-acyl groups to substrate proteins. Indeed the amino acid sequence of PIZ contains a consensus motif HxxxDG commonly found for the catalytic site of acyltransferases (Figure 7). Characterised enzymes from the same group include a deacetylvindoline 4-O-acetyltransferase (DAT) from *Catharanthus roseum* performing the last step in vindoline biosynthesis and an anthranilate N-hydroxycinnamoyl/benzoyltransferase (HCBT1) from *Dianthus caryophyllus* performing the first step in phytoalexin biosynthesis [46], [47] (Figure 7). In Arabidopsis, PIZ is a member of the BAHD family of acyltransferases consisting of more than 60 proteins involved in various physiological processes. The DFGWGKP motif close to the C-terminus is well conserved among BAHD acyltransferases [8], [48] but this sequence is only partially conserved in PIZ (Figure 7). The Arabidopsis genome does not appear to encode other proteins highly homologous to PIZ but a small family of three proteins, encoded by *At5g41040*, *At5g63560* and *At1g65450*, shows 29–31% identity and 48–50% similarity to PIZ (Figure 7). All of these proteins are uncharacterised but the protein encoded by *At5g41040* is closely related to a BAHD feruloyl-CoA transferase in suberin biosynthesis in potato [39], [49], [50].

### Heterologously Expressed PIZ Proteins Convert BL, CS and TY to the Corresponding Lauroyl Esters

**Figure 8:**
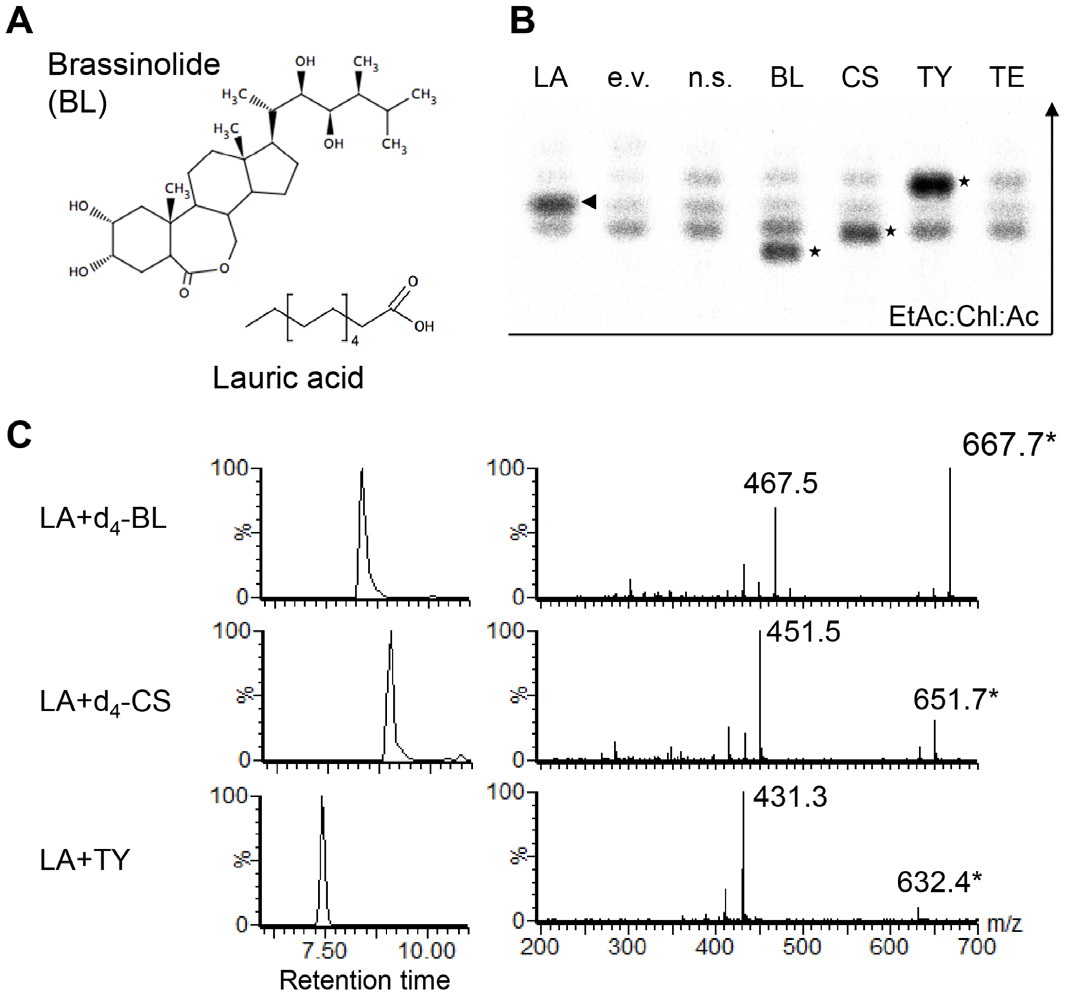
PIZ Converts BRs to the corresponding acylated products *in vitro*. (**A**) Structures of BL and ^14^C-lauric acid-CoA (LA-CoA). (**B**) Detection of ^14^C-labeled products extracted with ethyl acetate and separated by silica-gel thin layer chromatography. An asterisk marks the respective specific product for the applied substrates, BL, CS and TY, after 2 h of reaction at room temperature. An arrowhead marks free lauric acid (LA). e.v., empty vector control, n.s., no BR substrates. (**C**) LC-MS/MS analysis of the acylated products. Products were extracted from silica-gel after TLC and analysed on LC-MS/MS. Enzyme reactions were carried out using non-labelled LA and d_4_-BL, d_4_-CS or non-labelled TY. Predicted pseudo-molecular ions ([M+H]^+^) for the acylated products are shown with an asterisk.

Several studies have shown that BRs can be acylated in plants and for example, acylated esters of teasterone (TE) have been isolated from lily anthers or cell culture [51], [52]. To explore the possibility that PIZ acylates BRs in Arabidopsis, we tested a range of commercially available candidates for PIZ substrates in an *in vitro* assay. We expressed the full-length PIZ proteins, fused with His-tag on its N-terminus, in *E. coli* and purified them using the Ni-NTA column. Previous feeding experiments with 3,24-epibrassinolide suggest that the *β*-configuration of the C-3 hydroxy group of the substrate is essential for the acylation [51], [52], thus we included BL, CS and TY with C-3 in α-configuration, TE with C-3 in the *β*-configuration, and precursors with a *β*-configuration of the C-3 hydroxyl group. As a potential acyl-substrate, we chose radioactive labelled ^14^C-lauric acid-CoA as laureate-teasterone occurs naturally and it has a fatty acid chain of intermediate length (Figure 8A). For the identification of potential products, we extracted less polar fractions from 50 µL *in vitro* reaction mixture by ethyl acetate, separated potential products by thin layer chromatography (TLC) and detected spots containing ^14^C-LA by autoradiography.

After incubation of the reaction mixture containing PIZ proteins, BL and ^14^C-lauric acid-CoA for 2 h, we detected a band with an R_f_ value of 0.62 (Figure 8B). This band was not present in our negative control in which we used protein samples isolated from *E. coli* containing an empty vector or in a reaction without BR substrates (Figure 8B). In addition, products with R_f_ values of 0.67 and 0.78, respectively, accumulated specifically in the reactions containing CS and TY while we detected several other non-specific bands with similar R_f_ values. In contrast, addition of TE to the reaction at concentrations of 1.5 µM, 50 µM or 100 µM did not lead to a detectable product formation specific for PIZ (Figure 8B). Analysis of the putative product spots by LC-MS/MS detected compounds with a mass corresponding to the calculated molecular weight of the respective LA-ester ions of those substrates (with deuterium labelled BRs [LA-d_4_-BL+H]^+^: *m/z* 667.488 [LA-d_4_-CS+H]^+^: *m/z* 651.511 and unlabelled TY [LA-TY+H]^+^: *m/z* 631.376) as well as a major peak with a mass corresponding to the [BR-OH+H]^+^-ion lacking the LA moiety (*m/z* for d_4_-BL: 467.322, d_4_-CS: 451.346, TY: 431.259) (Figure 8C) that were absent in control spots sampled from corresponding areas of the separated reaction products after incubation without PIZ or a different substrate, respectively.

We also tested the preference of PIZ for fatty acid-CoA esters of different chain length in a competition assay. The addition of cold myristoyl-CoA ester (C14∶0) at a concentration of 20 µM instead of cold LA-CoA (C12∶0) almost completely diminished the formation of the radioactive LA-BL product band in the TLC while 20 µM Acetyl-CoA did not compete with 2 µM^ 14^C-LA-CoA (data not shown). These data suggest that the aliphatic tail of the CoA esters converted by PIZ is not restricted to lauric acid but needs a chain length of more than two carbon atoms with an expected preference of medium and long chain fatty acid moieties.

### PIZ is Dispensable for Organ Growth Under Standard Growth Conditions

**Figure 9:**
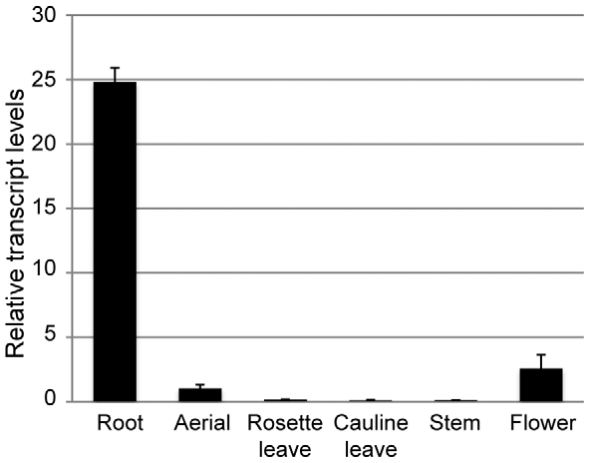
PIZ is expressed in various tissues in Arabidopsis. Transcript levels of *PIZ* in roots (Rt), aerial tissues (Ae), rosette leaves (RL), cauline leaves (CL), stems (St) and flowers (Fl) from wild-type plants. Mean values of three biological replicates are normalised to *ACT2*.

According to the publically available microarray data (eFP browser, http://bar.utoronto.ca/welcome.htm), the *PIZ* gene is expressed in various organs including leaves, roots and flowers but its expression level is generally low under standard growth conditions. Our quantitative RT-PCR experiments also confirmed the weak *PIZ* expression in aerial tissues and flowers as well as relatively strong expression in roots (Figure 9). To test whether the *PIZ* expression is modified by different BR levels, we examined the *PIZ* transcript level in *bri1-301* and *det2-1* roots in which endogenous BR levels are increased and decreased, respectively, compared to wild-type. We did not find statistically significant difference in *PIZ* mRNA levels, however, between wild-type and these mutants (data not shown). Using public coexpression analysis tools (ATTED-II, version 6.0, http://atted.jp), we also looked for genes that show similar expression patterns with *PIZ* but we did not find any other known BR-related genes co-expressed with *PIZ*.

To investigate the physiological role of *PIZ in vivo*, we obtained two T-DNA insertion lines *piz1* (SALK_123922) and *piz2* (SALK_137638) (Figure S3) and followed their development under our common growth conditions. However, when these mutants were grown on soil under short-day or long-day conditions, we did not detect any obvious growth defects (data not shown). Also roots grown on MS medium did not show a developmental phenotype differing from wild type. As this trend is common for enzymes in BR metabolism [22], [53], we also generated double and triple knock-out mutants with *BAS1* and *SOB7*, two genes with increased expression level in the *35S:PIZ* background. Although we could reproduce the *bas1* and *bas1 sob7* developmental phenotypes such as enlarged leaves and siliques, early flowering and longer hypocotyls in etiolated seedlings [53], additional loss of *PIZ* in *bas1*, *sob7* or *bas1 sob7* did not enhance any of those phenotypes (data not shown).

## Discussion

The metabolism, *i.e.* the removal of an active compound from cells, is a topic still understudied in phytohormone biology. As BR levels are exceptionally low in plants [54], [55], quick removal of excess BRs and their precursors might be of particular importance for the fine tuning of BR signalling. The biochemical analysis of BR metabolites in various plant tissues has revealed a large variety of possible modifications that can inactivate BL and CS [56] but we are still far from understanding how these modifications are mediated in plants. In this study we have identified a new BR metabolic enzyme. Our data show that PIZ metabolises physiologically relevant BRs *in vitro* and its overexpression in Arabidopsis leads to growth retardation similar to plants with reduced BR levels.

### PIZ Produces an Acylated form of BRs

In an *in vitro* enzymatic assay using radioactively labelled LA-CoA, PIZ converts BL, CS and TY to the corresponding lauric acid-esters. Consistent with these data, the level of BL, CS and TY is moderately to strongly reduced in *35S:PIZ* plants. Interestingly, however, only the application of BL and CS, but not TY, rescued the *35S:PIZ* phenotype in our spray experiments. Further *in vitro* analysis would be necessary to determine the kinetic properties of the PIZ reaction and its preferred substrates.

In BR biosynthesis, the *β*-configuration of the C3-hydroxy group is converted to the α-configuration in the two-step reaction from TE to TY via the 3-dehydroTE intermediate. In *Arabidopsis*, this step is suggested to be performed by the product of *CYP90D1* [7] which together with *ROT3*, is strongly induced in *35S:PIZ* background. Regarding the stereospecific preference for the substrate BRs, our results were slightly surprising because PIZ acylates only BL, CS and TY that have α-configuration of the hydroxy group at position C3. No endogenous fatty acyl-BRs have been detected so far in Arabidopsis and only endogenous BR-esters identified from several other plant species are TE-3*β*-myristate (C14∶0) and TE-3*β*-laureate (C12∶0) (reviewed in [56]). Previous feeding experiments with 24-EpiBL and 24-EpiCS with either α or *β* orientation of the hydroxy group at position C3 also showed a stereospecific selectivity for *β*-configuration because substrates with α-configuration were not converted to the corresponding fatty ester when applied to cell suspension culture of *Ornithopus sativus* [51], [52]. These data suggest that intermediates from earlier steps in BR biosynthesis up to TE possess the configuration expected to be acylated but we did not detect acylation of TE in our *in vitro* assay. Our data suggest that BRs with α-configuration are also acylated in Arabidopsis and we predict that identification of these types of BRs has been extremely difficult since those acylated BRs might be present in only small fractions *in vivo* and they are not detected by common methods which tend to focus on free BRs.

Future studies will also have to reveal the site of acylation by PIZ. The enzymes involved in BR metabolism, such as BAS1 and SOB7, so far act exclusively on the side chain and PIZ might also acylate those hydroxy groups or glycosides. Overexpression of a glycosyltransferase UGT73C6 leads not only to the production of a BL-23-glucoside but to a BL-23- malonylglucoside ester with the dicarboxylic acid malonate added to the glucosyl moiety [57]. However, the conversion of the active BRs and TY but not TE implies the importance of the C3 hydroxy group in α-configuration and therefore this moiety might be the target site or an allosteric factor for the enzyme-substrate binding. We do not exclude the possibility that PIZ targets other sites. In animals, esters of steroid hormones that are different from TE esters are common and several compounds are esterified at the 17*β*-position [58] or at other side chains [59] despite possessing a hydroxy group at position C3 (reviewed in [59]).

Structural studies and binding assays of BAS1 and SOB7 show preference of BAS1 for CS and BL while SOB7 prefers TY and does not bind to BL at all [60]. Despite the 35% amino acid sequence identity between these proteins, structural modelling revealed that SOB7 is an atypical cytochrome P450 and its substrate is inserted in a different orientation compared to BAS1 [60]. According to the lack of obvious homologues and its degenerated DFGWGKP motif, PIZ might not be a typical acyltransferase and similar structural studies on PIZ proteins would be also informative to bring further mechanistic insights into how PIZ modifies BRs.

### Physiological Roles of PIZ *in vivo*

The modification of BRs is usually associated with their inactivation (reviewed in [56]). Compared to the strong growth phenotypes of *35S:PIZ* plants, however, changes in active BR levels were relatively mild. This is surprising since strong mutant phenotypes often correlate with much stronger reduction in BR levels, e.g. down to 10% in *det2* [28]. Our data are, however, consistent with previous observation that a reduction of CS levels only to 85% leads to strong developmental defects in *cyp85a* (*br6ox*) mutants [11]. Notably BL levels have not been measured for many BR mutants due to its very low abundance, thus it is difficult to assess how general these apparent discrepancies are. BRs are thought to act locally within the tissue [61] and we suspect that *PIZ* overexpression may cause stronger down-regulation of BRs only in a subset of cells where BRs primarily function. Such local changes might be masked in our bulk analysis. Alternatively, acylation may also have some other inhibitory effects. In animals an acyl-substitute at the C3*β*-hydroxy group of cholesterol makes it an excellent substrate for the C20,22-side chain cleavage but depending on the length of the fatty acid moiety, it can also act as an inhibitor for this reaction [62]. It is possible, therefore, that acylated BRs block, for example, enzyme activities during later steps in BR biosynthesis. It is also known that acylation of steroid hormones hinders their diffusion through membranes and alters their binding to receptors [59]. Active BRs are produced in the endoplasmatic reticulum and probably they reach the apoplast via the vesicle trafficking. How BRs move within cells or across tissues has not been resolved yet but their movement or receptor binding might be interfered by acylation.

While overexpression of *PIZ* causes strong phenotypes in vegetative growth, we did not detect any apparent growth phenotypes in two loss-of-function *piz* mutants. The *piz bas1* double or *piz bas1 sob7* triple mutants showed published alterations in growth phenotypes but significant proportions of the phenotype were caused by the *bas1* mutation. These data suggest a major role for BAS1 in development and we speculate that PIZ might have more specific or redundant functions. A recently published study has uncovered another BAHD acyltransferase, BIA1, implicated in the inactivation of BRs in Arabidopsis [8]. Although biochemical activities of BIA1 as an acyltransferase have not been demonstrated, it is indeed possible that acylation of BRs is mediated by multiple acyltransferases and lack of PIZ is still masked by other redundant proteins. As *Arabidopsis* seeds are rich in oil, acylation might be more prominent during germination. During germination of radish seeds, a shift occurs from CS to BL, implying that BL has a role in germination, especially in shoot extension [63]. As natural BR acylation *in planta* has only been detected for teasterone, an alternative acyltransferase is expected to be involved in developmental processes like pollen tube elongation.

Some enzymes in BR metabolism are known to have a broad range of substrates and PIZ may also acylate additional substrates *in vivo*. A group of UDP-glycosyltransferases including UGT73C5 recognizes a range of aglycons including plant hormones and fungal mycotoxins *in vitro*. Production of BL-23-O-glycosyl in feeding experiments is completely diminished in Arabidopsis transgenic lines with silenced *UGT73C5* expression, implying a role of this enzyme in modifying BL [22]. UGT73C5 also converts a toxin deoxynivalenol and related compounds from *Fusarium*, and overexpression of the *UGT73C5* gene in Arabidopsis leads to higher resistance against this fungus. These data suggest that UGT73C5 may have dual functions in BR metabolism and fungal resistance [64]. It is also known that AtST4a, a sulfotransferase involved in BR metabolism, catalyses only BRs while other members of the same clade convert many other substrates including human estrogenic steroids and they function, for example, in detoxification [65], [66]. It is therefore possible that PIZ functions under some stress conditions such as pathogen attack or in the metabolism of xenobiotics.

## Materials and Methods

### Chemicals, Plant Materials and Growth Conditions

Phytohormones were purchased from Brassino Co. Ltd (Japan) or Sigma-Aldrich (Japan) as salts and unless described otherwise, dissolved in DMSO as at least 100× stocks for experiments. Radioactive lauroyl Coenzyme A [lauroyl-1-^14^C] was purchased from American Radiolabelled Chemicals Inc. (USA) in sodium acetate buffer (specific activity 1.85–2.22 GBq/mmol).

All plants used in this study were in Columbia (Col-0) ecotype. The *35S:PIZ* line (F23131) was isolated from the FOX mutant collection [12] and two *piz* mutant alleles (SALK_123922 and SALK_137638) were obtained from the ABRC Stock Center. The *det2-1*, *br6ox1 br6ox2* (*cyp85a1 cyp85a2*), and *ga3ox1 ga3ox2* mutants were described previously [28], [29], [30]. The *bas1* [53] and *bri1-301* mutants [67] were provided by Michael Neff and Guang Wu, respectively.

An insertion of the *At4g31910* cDNA within the FOX vector pBIG2113SF of original F23131 plants was detected by sequencing the PCR products amplified with vector-specific oligonucleotides (GS15 3′-ATTACATTTTACATTCTACAACTACATCT-5′ and GS16 3′-CAAATGTTTGAACGATCGGGGAAAT-5′). In further generations, the presence of *At4g31910* cDNA was confirmed by PCR using GS15 and a gene specific reverse primer (4g31910SR1 3′-ATCGTTACACCGGATGTTGAG-5′). The *bri1-301* genotype was confirmed by PCR using the primer pair Bri301_F and Bri301_R [67].

The *Col-0, piz1, bas1 and piz1 bas1* lines were isolated from a cross between *bas1 sob7* [53] and *piz1* (SALK_123922) using gene specific primers, 922F1 3′-ATGTTAATGGCGACACGTATCG-5′ and 922R1 3′-ACAGGACGTACTTAATAACAATCG-5′ for *PIZ1* and as previously reported for *BAS1* [53], or a combination of these primers and the left border primer for the SALK T-DNA insertion pBI LB61 3′-GTAAAACGACGGCCAGT-5′.

Unless stated otherwise, seeds were imbibed for 3 days at 4°C either in water or on soil and grown either on Murashige-Skoog (MS) medium (pH 5.7, 1% sucrose and 0.5% phytogel) or on a 1∶1 mixture of soil Supermix A (Sakata, Japan) and vermiculite (VS Kako, Japan) in continuous light (40–60 µE m^−2^ sec^−1^) at 22°C.

### Analysis of Developmental Phenotypes

Phenotypes of leaves from soil-grown plants were documented using a digital camera (Fujifilm FinePix F40) or an image scanner (EPSON, GT-X970) with appropriate size standards. Petals (n>35) were removed from fully opened flowers and photographs were taken using stereomicroscope (Leica M165 FC). About 5 mg seeds from three batches per line were weighted and counted to estimate seed weight. Size measurements were performed with ImageJ 1.40 g software (National Institutes of Health, http://rsb.info.nih.gov/ij). Mature first and second true leaves from 3-week-old plants (n = 20) were harvested and the area, length and width of leaf blades as well as the length of petioles were quantified. To examine leaf cell size, five leaves of intermediate size were fixed overnight with acetic acid:ethanol (1∶1), de-hydrated by ethanol solution of ascending concentration, and cleared in chloral hydrate:H_2_O:glycerol (8∶2:1 w/w). Pictures of three corresponding areas per leaf were taken using a differential interference contrast microscope (Olympus BX51) and the area of mesophyll cells was measured using ImageJ (n = 300). To estimate the number of cells per leaf, the average number of cells in four areas of four leaves was extrapolated to the average blade area. For ploidy analysis, first and second true leaves of 2–3 plants were pooled, chopped and subjected to flow cytometry analysis using the ploidy analyser PA-I (Partec) up to a count of 5000–10,000 nuclei. Root length of seedlings grown on vertical MS medium was recorded using an image scanner and analysed with ImageJ.

### Sequence Analysis

The amino acid sequences of PIZ and related proteins from TAIR or Genbank were aligned using ClustalW (http://align.genome.jp/) in the multiple alignment mode with a gap opening penalty of 10.0 and a gap extension penalty of 0.05. The phylogenetic tree was created using the same program. Prediction of protein function was performed using InterProScan (http://www.ebi.ac.uk/Tools/InterProScan/) from EMBL-EBI [68].

### Complementation Assay

For complementation of the *35S:PIZ* dwarf phenotype, soil-grown plants were sprayed with BL of 0, 0.01, 0.1 or 1 µM, dissolved in water supplemented with 0.1% Tween 20, every fourth day from the fourth day after sowing until ripening. Equal covering of aerial plant tissue with droplets was monitored by eye. In addition, seedlings were grown on MS plates supplemented with 0, 0.01, 0.1 or 1 µM of BL or CS for 3 weeks. For GA_4_ treatment, seeds were imbibed in 50 µM GA_4_ in water, rinsed three times, and sown on MS plates with GA_4_ of 0, 0.01, 0.1 or 1 µM.

### Heterologous Expression of PIZ in *E. coli* and *in vitro* Assay

The *PIZ* cDNA was amplified from the FOX plasmid with gene specific primers and cloned into the pET16b vector via *Nde*I and *BamH*I restriction sites introduced within the primers. The protein was expressed in *E. coli* BL21 DE3 Lys3 cells, extracted with BugBuster® Protein Extraction Reagent (Novagen), and purified on Ni-NTA agarose (Qiagen) via the N-terminal His-tag encoded within pET16b. Purity of the protein in the elution fractions was monitored by SDS-PAGE and Coomassie Brilliant Blue staining.

The elution fractions highly enriched in PIZ were used for the radioactive enzyme assay. To screen for potential substrates, reactions were carried out in 100 µL volumes containing 20 µM cold LA-CoA, ∼10,000 dpm ^14^C-LA-CoA (∼1 µM), 1.5 µM to 100 µM of BR substrate, 5 mM dithiothreitol (DTT) and 2–5 µL of enzyme fraction in 0.1 M sodium phosphate buffer. After 2 h and 24 h at 25°C, 45 µL aliquots were subjected to extraction. The reaction was stopped with 100 µL hydrochloric acid and 800 µL water. The lipophilic substrates were extracted twice with 1 mL ethyl acetate and the organic fractions were combined and vaporised under nitrogen. The dried fractions were resuspended in 20 µL methanol and separated on silica 60 thin layer chromatography (TLC) plates with ethylacetate:chloroform:acetate 70∶30:1. The radioactivity in spots was detected after overnight exposure to a Storage Phosphor Screen (BAS-IP MS2040) using a Typhoon™ FLA 7000 Biomolecular Imager (GE Healthcare).

To analyse the products by LC-MS/MS, we incubated recombinant PIZ proteins with d_4_-BL, d_4_-CS or TY. The reaction products after 24 h at 25°C were separated on silica gel TLC as described above and putative acylated BRs in spots on the TLC plate were scraped and collected. Acylated BRs were eluted with water-saturated ethylacetate from the scraped silica gel powder and they were then subjected to LC-MS/MS analysis using a quadruple/time-of-flight tandem mass spectrometer (Q-tof Premier, Waters) and an Acquity Ultra Performance liquid chromatograph (Waters).

### Microarray Analysis

Microarray experiments were performed using 3-week-old plants of a segregating *35S:PIZ* line grown on soil under continuous light at 22°C. Arial tissues of 4 to 5 WT or *35S:PIZ* with a clearly distinguishable phenotype were combined for each sample. Total RNA was extracted with the RNeasy Plant Mini Kit (Qiagen) from three biological replicates for each genotype and 10 µg were subjected to the Affymetrix ATH1 chip assay. Microarray data were analysed using GeneSpring Vers.11 (Agilent Technologies). Expression levels were normalized using the MAS 5.0 algorithm and quality control was performed with GeneSpring 11 using standard settings.

### Quantitative PCR

For quantitative PCR of WT and *35S:PIZ*, aerial tissues of 17-day-old plants grown on soil were collected for two independent samples. To quantify the endogenous *PIZ* transcript level, aerial tissues, rosette leaves, cauline leaves, flowers and roots were harvested from wild-type plants. RNA was extracted with the RNeasy Plant Mini Kit (Qiagen) and subscribed to cDNA using the SuperScript III Reverse Transcriptase Kit (Invitrogen). Real time PCR was performed with three technical replicates using the SYBR green Thunderbird Kit (Toyobo), the Mx3000 cycler and MxPro 4.10 Software (Stratagene). Following primer pairs were used to amplify the *PP2AA3* (*At1g13320.1*) or *ACT2* (*At3g18780*) gene used to normalise the expression levels and other BR-related genes: 1g13320Rf2 3′-GACCAAGTGAACCAGGTTATTGG-5′ and 1g13320Rr2 3′-TACTCTCCAGTGCCTGTCTTCA-5′ for *PP2AA3,* ACT2F 3′-CTGGATCGGTGGTTCCATTC-5′ and ACT2R 3′-CCTGGACCTGCCTCATCATAC-5′ for *ACT2*, 4g31910SF1 3′-CATTCACACACACCCACATT-5′ (Figure 5) or 4g3190SF2 3′-ATGTGCTCCTCCTTTGAGTTTC-5′ (Figure 3) and 4g31910SR1 3′-ATCGTTACACCGGATGTTGAG-5′ for *PIZ*, BAS1FW3 3′-CCAAGGACCATGTCGTTAAGC-5′ and BAS1RV2 3′-CCTGAAGTATAGCAAGATTCTGACC-5′ for *BAS1*, SOB7FW2 3′-GTCAGCAAAGAACTAAAGAATCC-5′ and SOB7RV2 3′-GCTCGAATAGCAAGGAGACC-5′ for *SOB7*, UGT_q_fw2 and UGT_q_rv2 [64] for *UGT73C5*, primer sets published in [41] for *CYP90D1,* and CPD-realtime F 3′-GCACTTTCAACCCTTGGAGA-5′ and CPD-realtime R 3′-GAACCCAACTGAAGCCTGTC-5′ for *CPD,* ROT3-realtime F 3′-AAAGGTTACTTAATACCGAAAGGATG-5′ and ROT3-realtime R 3′-CCATTAATTCTGTCCCATCTCC-5′ for *ROT3,* and BR6ox2-realtime F 3′-TGGCATTTTTATCATCGTTGT-5′ and BR6ox2-realtime R 3′-TTCTTGCTTGGACTCCACTG-5′ for *BR6ox2*.

### Analysis of Endogenous BR Levels

Seedlings from respective lines were grown in parallel on soil and aerial tissues were collected from 16 to 18-day-old plants. Harvested materials were immediately frozen in liquid nitrogen and stored at −80°C. Endogenous levels of BL and CS were quantified by LC-ESI-MS/MS as previously described [69] and endogenous levels of BR precursors were analysed by GC-MS as described before [70].

## Supporting Information

Figure S1 **The dwarf phenotype of** ***35S:PIZ*** **seedlings is complemented by exogenous BL or CS but not by GA_4_.** Plants were grown on MS plates supplemented with indicated concentrations of BL, CS or GA_4_. For BL and CS treatment, strong growth recovery of BL biosynthesis mutants *br6ox1 br6ox2* and *det2-1* are shown as positive controls. For GA_4_ treatment, the growth recovery of GA biosynthesis mutant *ga3ox1 ga3ox2* is shown as a positive control.
(TIF)

Figure S2 **Possible position of PIZ in BR metabolism.** Brassinosteroid biosynthesis pathway (modified from [71]). Acylation steps of PIZ, suggested by *in vitro* enzymatic assays, are shown in black arrows. Grey arrows indicate potential further acylation steps. Graphs indicate reduction of the respective intermediates in *35S:PIZ* as in Figure 6.
(TIF)

Figure S3 **The structure of the** ***PIZ*** **gene.** The black boxes represent exons and lines represent introns. The T-DNA insertion sites for *piz1* (SALK_123922) and *piz2* (SALK_137638) are indicated by arrowheads.
(TIF)
