# Supplementary figures and images for: Arabidopsis PIZZA Has the Capacity to Acylate Brassinosteroids
Source: PLoS One. 2012 Oct 5;7(10):e46805. doi: 10.1371/journal.pone.0046805 (PMC3465265; doi:10.1371/journal.pone.0046805)

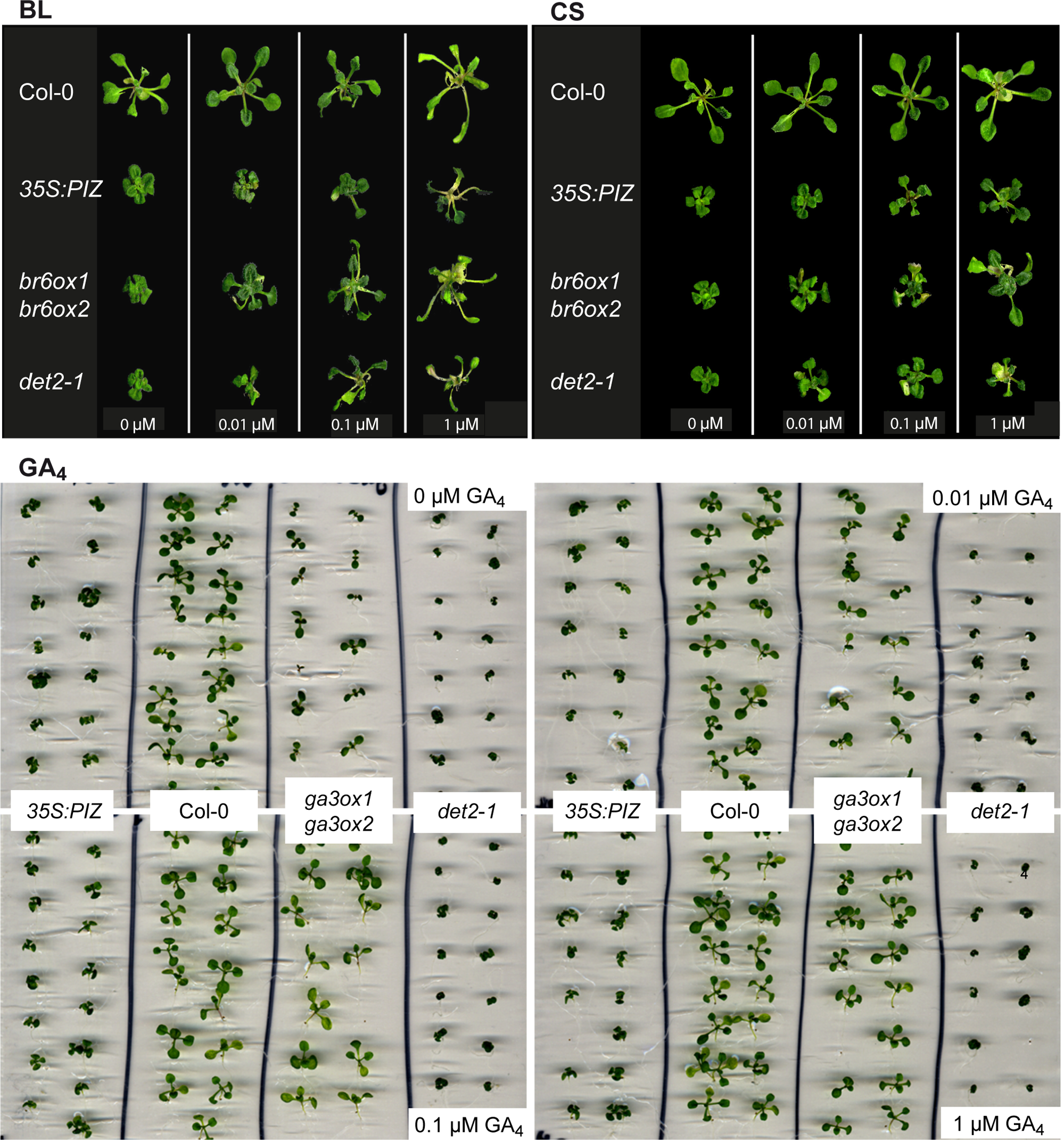

Supplement: Figure S1 — The dwarf phenotype of 35S:PIZ seedlings is complemented by exogenous BL or CS but not by GA4. Plants were grown on MS plates supplemented with indicated concentrations of BL, CS or GA4. For BL and CS treatment, strong growth recovery of BL biosynthesis mutants br6ox1 br6ox2 and det2-1 are shown as positive controls. For GA4 treatment, the growth recovery of GA biosynthesis mutant ga3ox1 ga3ox2 is shown as a positive control. (TIF) [file pone.0046805.s001.tif]

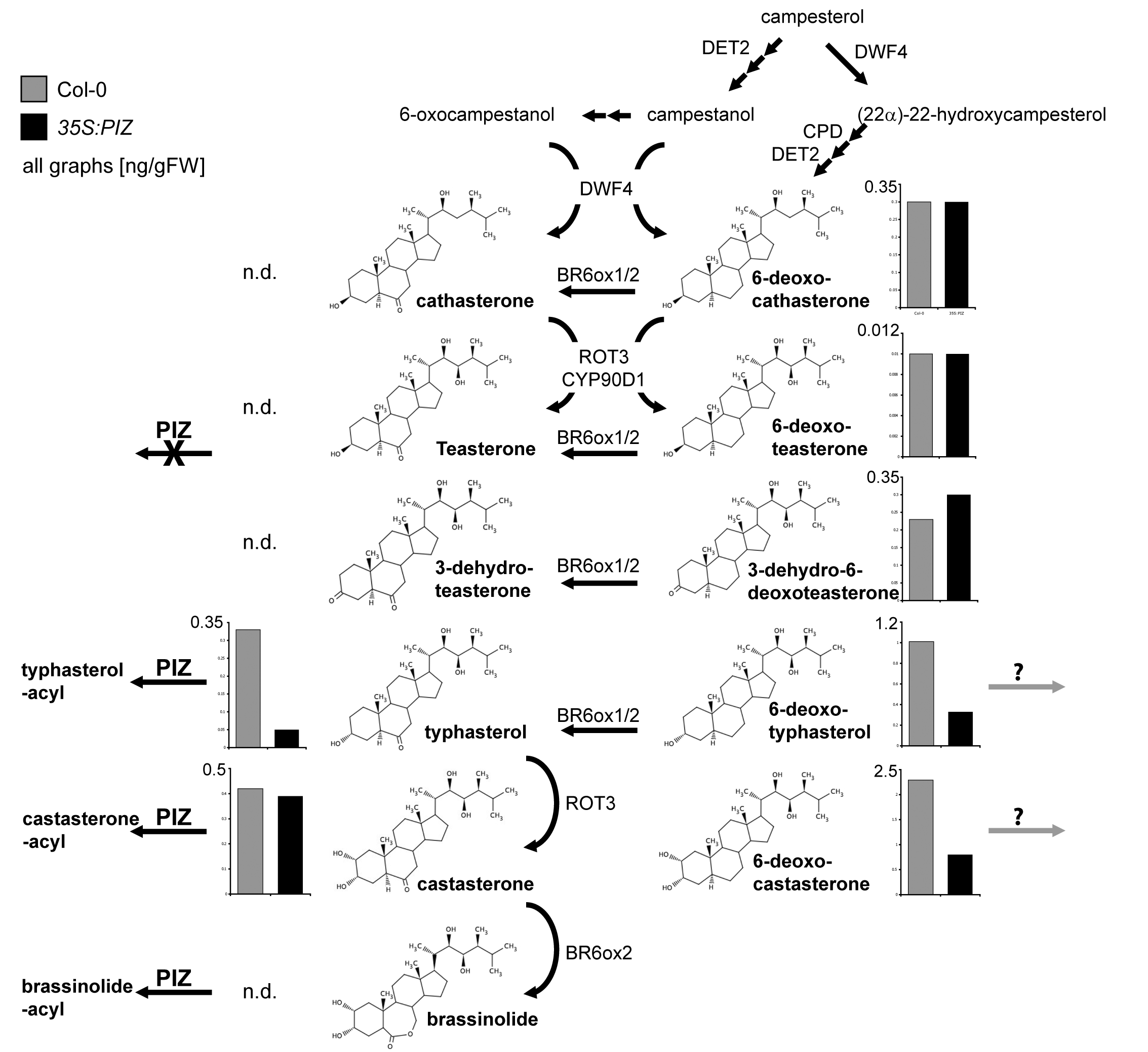

Supplement: Figure S2 — Possible position of PIZ in BR metabolism. Brassinosteroid biosynthesis pathway (modified from [71]). Acylation steps of PIZ, suggested by in vitro enzymatic assays, are shown in black arrows. Grey arrows indicate potential further acylation steps. Graphs indicate reduction of the respective intermediates in 35S:PIZ as in Figure 6. (TIF) [file pone.0046805.s002.tif]

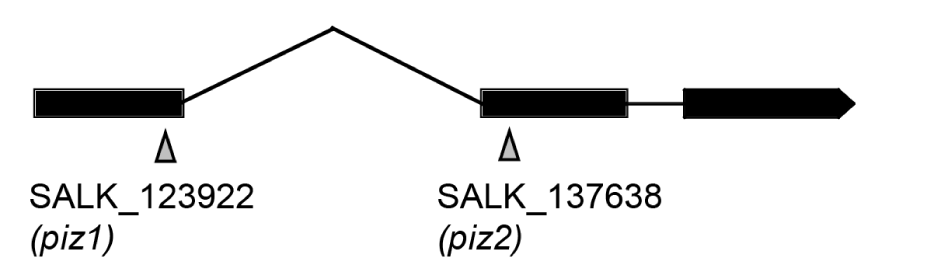

Supplement: Figure S3 — The structure of the PIZ gene. The black boxes represent exons and lines represent introns. The T-DNA insertion sites for piz1 (SALK_123922) and piz2 (SALK_137638) are indicated by arrowheads. (TIF) [file pone.0046805.s003.tif]
